# Intraoperative Incidence of Bacteremia During Surgical Management of Non-tubercular Spondylodiscitis: A Prospective Analysis

**DOI:** 10.7759/cureus.100495

**Published:** 2025-12-31

**Authors:** Bharat R Dave, Shivanand C Mayi, Arjit Vashishtha, Mahesh Sagar, Ajay Krishnan, Ravi Ranjan Rai, Mirant B Dave, Mikeson Panthackel, Amritesh Singh, Saurabh S Kulkarni, Yogenkumar Adodariya

**Affiliations:** 1 Spine Surgery, Stavya Spine Hospital and Research Institute, Ahmedabad, IND; 2 Spine Surgery, Stavya Spine Hospital and Research Institute, Ahmedhabad, IND

**Keywords:** bacteremia, intraoperative blood culture, pyogenic spondylodiscitis, spinal infection, vertebral osteomyelitis

## Abstract

Introduction

Non-tubercular spondylodiscitis is a serious spinal infection that can lead to significant morbidity. The potential for bacteremia during surgical manipulation of infected disc material has not been well studied. This prospective study aimed to evaluate the incidence of bacteremia during the surgical procedure in patients with non-tubercular spondylodiscitis.

Methods

A single-center, prospective study was conducted from August 2019 to May 2020, enrolling 28 consecutive patients with biopsy-proven or MRI-suspected non-tuberculous vertebral osteomyelitis who underwent surgery. Preoperative blood, urine, and throat swab cultures were performed after a 48-hour antibiotic-free interval for patients who had been on prior antibiotics. Intraoperative venous blood cultures were collected immediately after disc preparation but before antibiotic administration. Surgical procedures included cervical discectomy/corpectomy, thoracic laminectomy with fixation, and lumbar Transforaminal Lumbar Interbody Fusion (TLIF). Culture results from blood, disc material, and other samples were analyzed.

Results

Intraoperative blood culture was positive in three patients (12.5%), with one patient showing the same organism preoperatively. Five patients had growth in disc material cultures; only one matched preoperative and perioperative blood cultures with methicillin-resistant *Staphylococcus aureus *(MRSA). Other samples showed variable pathogen growth without matching patterns. The overall intraoperative bacteremia rate was low, suggesting limited bloodstream entry during surgical manipulation.

Conclusion

Surgical manipulation of infected disc material in non-tubercular spondylodiscitis patients was not associated with a significant increase in bacteremia. However, a small sample size and only a few positive sample results are limitations of the study to conclude, and further multicentric studies with larger cohorts and multiple perioperative blood culture samples are warranted for more definitive conclusions.

## Introduction

Spinal infections are classified aetiologically as pyogenic, granulomatous (including tuberculous, brucella, and fungal), and parasitic [1]. Spondylodiscitis comprises vertebral osteomyelitis, spondylitis, and discitis, and if left untreated, may progress to epidural abscess, meningitis, spinal cord abscess, or subdural empyema [1,2]. This condition has longstanding historical roots, with evidence of spinal tuberculosis documented in ancient human skeletons [3]. Pyogenic vertebral osteomyelitis was first described by the French physician Lannelongue in 1879, and Kulowski published further literature on pyogenic vertebral infections in 1936 [4,5]. Over time, the introduction of surgical advances, improved radiological techniques, and the discovery of antimicrobial therapies have altered the approach to spinal infection, yet significant morbidity remains [6].

Spondylodiscitis is the predominant form of hematogenous osteomyelitis in individuals older than fifty, accounting for 3-5% of all cases [7,8,9]. The incidence of vertebral infections has risen as the susceptible population grows and diagnostic tools improve [7,10,11]. Factors contributing to this increase include intravenous drug use, more frequent healthcare-associated infections, exposure through spinal surgery, and a higher proportion of immunosuppressed and elderly patients [11-14].

Hematogenous pyogenic spondylodiscitis most commonly affects the lumbar spine, followed by the thoracic and cervical regions (58%, 30%, and 11%, respectively) [15]. Cervical lesions are seen more often in intravenous drug users, and multifocal involvement occurs in about 4% of cases [15,16].

Bacteria may enter the bloodstream (bacteremia) during various medical and dental procedures, and blood cultures can be used to identify them. Similarly, during a Transforaminal Lumbar Interbody Fusion (TLIF) procedure, which is done for stabilisation, debridement of necrotic tissue, and fusion with bone graft, handling of the infected disc tissue during discectomy and end-plate preparation can theoretically transmit the causative bacteria into the bloodstream. This surge of bacteremia could potentially be a source of systemic infection and postoperative sepsis, especially in immunocompromised patients. Additionally, haematogenous spread can give rise to another infection foci elsewhere in the body. 

Blood culture is a straightforward, cost-effective method for identifying the bacterial agents responsible for spondylodiscitis [17]. Its reported diagnostic yield ranges from 40% to 60% in clinically recognised cases, with about 25% to 59% of positive cultures pinpointing the causative organism [4,18]. We believe that manipulation of infected disc material during discectomy and endplate preparation while doing TLIF [19] may introduce bacteria into the bloodstream, detectable by intraoperative blood cultures collected after disc preparation.

The primary aim of this study is to assess the possible occurrence of bacteraemia in pyogenic spondylodiscitis patients undergoing surgery. Certain measures, such as the administration of prophylactic antibiotics, can prevent complications like postoperative sepsis. 

For this study, we created a null hypothesis that manipulating infected disc material during discectomy and endplate preparation during TLIF does not introduce bacteria into the bloodstream. 

## Materials and methods

This single-centre, prospective study was conducted at a tertiary care spine hospital from August 2019 to May 2020. Ethical approval was obtained from the Institutional Ethics Committee of Stavya Spine Hospital and Research Institute (approval number SSHRI/NS/PSD/BRD/28/072019) prior to initiation. Consecutive patients with either biopsy-proven or MRI findings suggestive of non-tubercular spondylodiscitis who were scheduled for surgery were enrolled after obtaining informed consent. Patients previously on antibiotic treatment were admitted, and antibiotics were withheld for 48 hours before preoperative blood, urine, and throat swab sample collection. Those with biopsy-confirmed tuberculous spondylodiscitis or MRI evidence of spinal tuberculosis were excluded.

Routine blood investigations necessary for determining surgical fitness were performed according to hospital protocol. In addition, blood, urine, and throat swab cultures were obtained preoperatively, one day before surgery, to screen for bacteraemia and identify possible infectious foci. The surgical procedures were done adhering to the standard protocols and were carried out without administering preoperative intravenous antibiotics. Cervical spine involvement was managed with an anterior approach; procedures included cervical discectomy or corpectomy, followed by placement of a bone graft or bone graft with a cage, and subsequent fixation with a plate and screws. For thoracic spine disease, treatment involved pedicle screw fixation, laminectomy, removal of infected disc material, and bone graft placement in the disc space or posterolateral region. Lumbar cases involved pedicle screw fixation, laminectomy, and either unilateral or bilateral facetectomy for the Transforaminal Lumbar Interbody Fusion (TLIF) procedure. The annulus was incised as planned, and sequentially sized curettes and reamers were used to prepare the interbody space. All the infected and necrotic disc material was removed and collected in a sterile leak-proof container using disc forceps, under all aseptic conditions, taking care that the tissue does not touch any skin area, and sent to the laboratory at room temperature. The endplates were prepared by scraping the endplate cartilage with curettes and continued until the endplates began to bleed. During disc preparation, a venous blood sample was collected for culture by the anaesthetist.

After strict sterile skin preparation with spirit, under all aseptic precautions, a new intravenous access cannula was utilised to facilitate intraoperative blood sample collection after disc preparation. Sixteen millilitres of venous blood were drawn and immediately transferred into culture media bottles, with eight millilitres added to an anaerobic (BD BACTEC Plus Anaerobic/F Culture Vial; BD, New Jersey, USA) and another eight to an aerobic (BD BACTEC Plus Aerobic/F Culture Vial) bottle. The culture bottles were gently inverted a few times to mix the blood with the culture media. After labelling, both specimens were rapidly sent to the laboratory at room temperature for processing. Intravenous antibiotics were administered after the collection of disc material and blood samples. The surgical procedure was completed by filling the disc space with either a bone graft, a cage, or both, and closing the wound over a suction drain. Antibiotic regimens were adjusted according to culture and sensitivity reports from preoperative or postoperative blood and disc material samples. The drain was removed within three to five days following surgery, and antibiotics were continued for six weeks - three weeks intravenously followed by three weeks orally, as per institutional and infection specialist protocols.

Positive culture from the intraoperatively collected blood sample is interpreted as a marker of the occurrence of bacteremia. 

Statistical analysis

Demographic details of the patients enrolled were documented along with their history and the affected region of the spine. All the preoperative (blood, throat swab, and urine culture) as well as intraoperative blood culture reports were collected and analysed. Microsoft Excel (Microsoft Corporation, Redmond, USA) was used for the documentation in tabular form and analysis of the collected demographic and culture reports data, along with the affected region of the spine. Demographic and quantitative variables were summarised as mean ± standard deviation, including range (minimum-maximum). 

## Results

**Figure 1:**
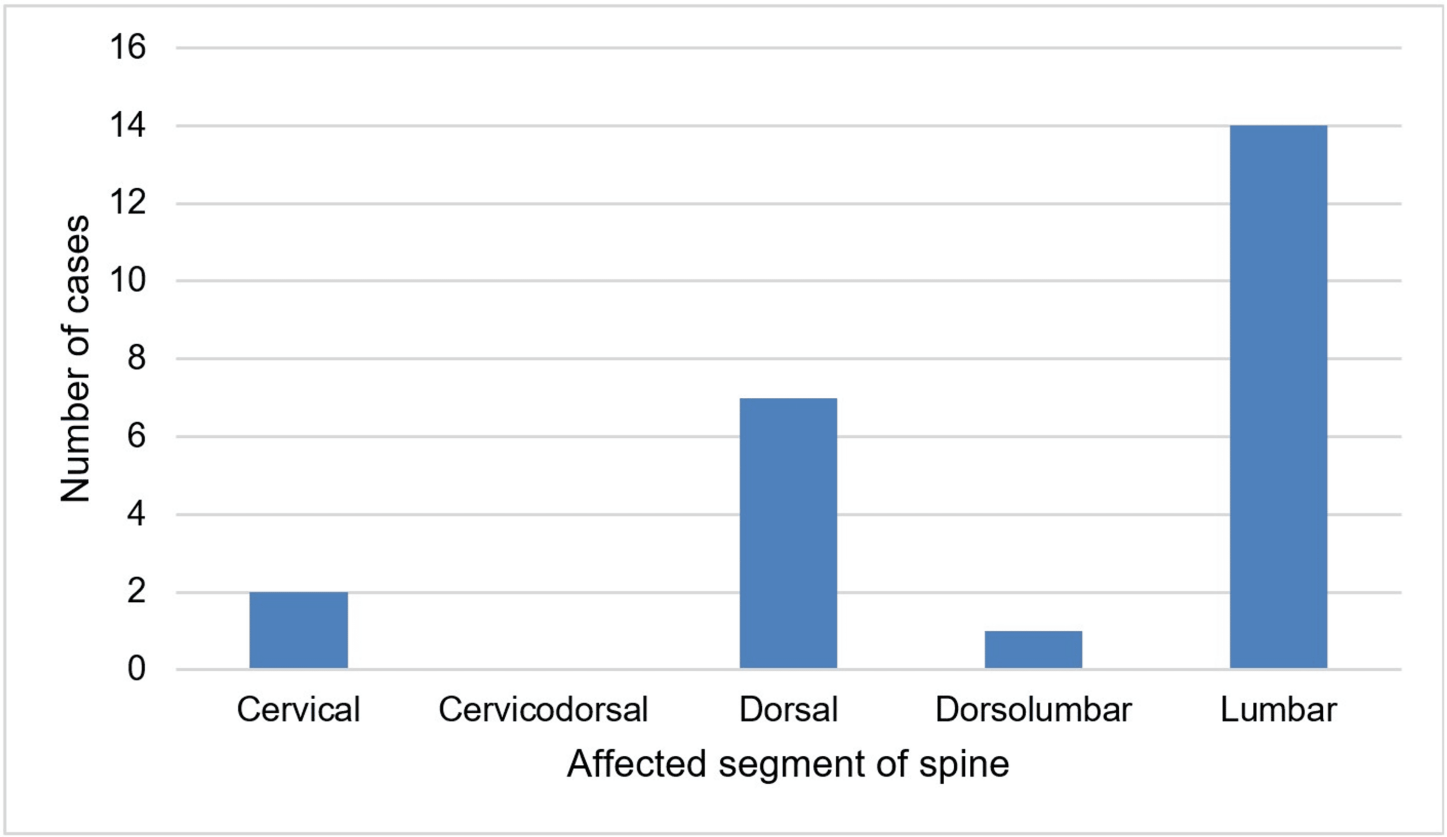
Segmental distribution of cases

In this study, a total of 28 consecutive patients who fulfilled the inclusion criteria were enrolled. Four patients were reported positive for tuberculosis by GeneXpert and thus were excluded from the analysis. Out of the remaining 24 patients, 15 were male, and nine were female. The age of the patients included in the study varied from 13 years to 70 years, with a mean age of 53.5 ± 13.7 years (Table 1). The lumbar spine (n=14) was the most common segment involved, followed by the dorsal spine (n=7). The segmental distribution of cases is given in Figure 1.

**Table 1:** Demographic data of the study population

Variable	Category/Measure	Value
Gender (n (%))	Male	15 (62.5%)
	Female	9 (37.5%)
	Total	24
Age (years)	Minimum	13
	Maximum	70
	Mean ± SD	53.5 ± 13.7

Intraoperative venous blood culture revealed growth in three patients (12.5% positivity rate, with Wilson's 95% CI from 4.3% to 31%). Notably, only one patient (patient number 6) had the same organism identified in their preoperative venous blood culture, as shown in Table 2.

**Table 2:** Culture growth pattern. *E. coli *= *Escherichia coli*; *S. aureus *= *Staphylococcus aureus*; *Klebsiella *spp = *Klebsiella *species; MSSA = methicillin-sensitive *Staphylococcus aureus*; MRSA = methicillin-resistant *Staphylococcus aureus*; MSSE = methicillin-sensitive *Staphylococcus epidermidis*; MRSE = methicillin-resistant *Staphylococcus epidermidis*; *Enterococcus *spp = *Enterococcus *species; *Candida *spp = *Candida *species; *Pseudomonas *spp = *Pseudomonas *species. *GeneXpert® nucleic acid amplification test by Cepheid India Pvt. Ltd., Bengaluru, India.

S. No.	Sex	Preoperative			Intraoperative			GeneXpert®*
		Urine Culture	Blood Culture	Throat Swab Culture	Disc Material Culture	Blood Culture	Histopathology	
1	M	E. coli	No growth	No growth	S. aureus	No growth	Chronic inflammation	Negative
2	F	E. coli	No growth	No growth	No growth	No growth	Chronic inflammation	Negative
3	F	No growth	No growth	No growth	No growth	No growth	Chronic inflammation	Negative
4	F	No growth	No growth	No growth	Klebsiella sp.	No growth	Chronic inflammation	Negative
5	M	No growth	No growth	MSSA	No growth	No growth	Chronic inflammation	Negative
6	F	No growth	MRSA	No growth	MRSA	MRSA	Chronic inflammation	Negative
7	M	No growth	No growth	No growth	No growth	No growth	Chronic inflammation	Negative
8	M	No growth	No growth	Klebsiella sp.	No growth	No growth	Chronic inflammation	Negative
9	M	No growth	No growth	No growth	No growth	No growth	Chronic inflammation	Negative
10	M	No growth	No growth	No growth	Enterococcus sp.	No growth	Chronic inflammation	Negative
11	F	No growth	No growth	No growth	No growth	No growth	Chronic inflammation	Negative
12	F	No growth	No growth	No growth	No growth	No growth	Chronic inflammation	Negative
13	M	No growth	No growth	No growth	No growth	No growth	Chronic inflammation	Negative
14	M	No growth	No growth	No growth	No growth	No growth	Chronic inflammation	Negative
15	M	No growth	No growth	No growth	No growth	No growth	Chronic inflammation	Negative
16	M	E. coli	No growth	Serratia marcescens	No growth	No growth	Chronic inflammation	Negative
17	M	No growth	No growth	MSSE	No growth	No growth	Chronic inflammation	Negative
18	F	No growth	No growth	E. coli	No growth	MRSE	Chronic inflammation	Negative
19	M	*E. coli *and* Candida sp.*	No growth	No growth	No growth	MRSE	Chronic inflammation	Negative
20	M	Candida sp.	No growth	No growth	No growth	No growth	Chronic inflammation	Negative
21	F	No growth	No growth	No growth	No growth	No growth	Consistent with Tuberculosis	Positive
22	M	Pseudomonas sp.	No growth	Normal Flora	S. aureus	No growth	Acute and chronic infection	Negative
23	F	No growth	MSSE	No growth	No growth	No growth	Chronic inflammation	Positive
24	F	No growth	No growth	Pseudomonas sp.	No growth	No growth	Consistent with Tuberculosis	Positive
25	M	Pseudomonas sp.	No growth	E. coli	No growth	No growth	Acute and chronic infection	Negative
26	M	No growth	No growth	Pseudomonas sp.	No growth	No growth	Chronic inflammation	Positive
27	F	E. coli	Enterococcus faecalis	No growth	No growth	No growth	Chronic inflammation	Negative
28	F	No growth	No growth	No growth	No growth	No growth	Chronic inflammation	Negative

**Figure 2:**
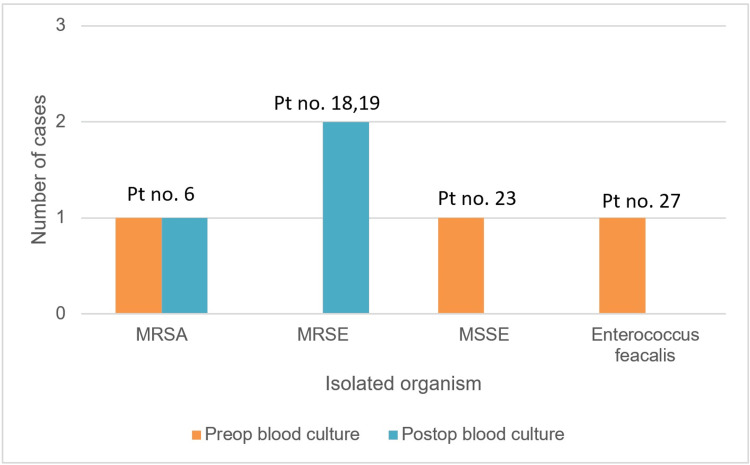
Blood culture growth pattern Pt no. = patient number, Preop = preoperative, Postop = postoperative, MSSA = methicillin-sensitive *Staphylococcus aureus*; MRSA = methicillin-resistant *Staphylococcus aureus*; MSSE = methicillin-sensitive *Staphylococcus epidermidis*; MRSE = methicillin-resistant *Staphylococcus epidermidis*

Out of the other two patients, one had no growth in the preoperative blood culture (patient number 19), and the other had *E. coli *(patient number 18), as shown in Figure 2. One patient out of these three (patient number 6) had the same organism grown on disc material culture, while the other two didn’t show any growth.

Preoperative throat swab culture showed growth of the pathogenic organism in eight patients and normal commensals in one patient. Preoperative urine culture showed growth of pathogenic bacteria in seven patients, with one of them showing mixed growth with *E. coli *and *Candida*, probably suggestive of urinary tract infection, which was not clinically relevant to this study, as no other culture report had positive growth of these organisms. Five patients had bacteria grown on their disc material culture, of which one patient had preoperative and perioperative blood culture positive, with all three samples growing methicillin-resistant *Staphylococcus aureus* (MRSA). The remaining four patients did not have any growth in blood samples (Table 3).

**Table 3:** Positivity rates of cultures

	Preoperative			Intraoperative	
	Urine Culture	Blood Culture	Throat Swab Culture	Disc Material Culture	Blood Culture
Positive Culture Growth	7	3	9	5	3
Positivity Rate	29.1%	12.5%	37.5%	20.8%	12.5%

GeneXpert® (nucleic acid amplification test by Cepheid India Pvt. Ltd., Bengaluru, India) for tuberculosis was positive in four patients, who were excluded from the analysis. Among them, one patient showed growth of methicillin-sensitive *Staphylococcus epidermis *(MSSE) in preoperative blood culture. Histopathological examination of disc material was consistent with tuberculosis (TB) findings in two patients, and the other two had chronic inflammatory features. Throat swabs and urine culture in the preoperative period showed the growth of organisms that did not match the blood or tissue culture growth reports.

## Discussion

Hematogenous spread is the most frequent route for spinal infections [18]. Primary sources may originate in the oral cavity, skin, respiratory tract, urinary system, gastrointestinal tract, or from infected implanted devices [18]. Iatrogenic bacterial inoculation accounts for approximately 14% to 26% of spinal infections [20]. In this study, however, only one patient (4.1%, n=1/24) had MRSA growth detected in preoperative blood culture, with the same organism identified in both infected disc material culture and intraoperative blood cultures, confirming hematogenous spread. The rest of the study group, who presented with different pathogenic growths in urine and throat swab cultures, did not have matching organisms in their preoperative or intraoperative blood samples, reflecting a lower likelihood of bacteremia during the surgical procedure.

In our study, all blood samples were obtained after stopping antibiotics for 48 hours, and the positivity rate was 12.5% ((n=3/24), Wilson's 95% CI from 4.3% to 31%), which is lower than previously reported results. Positive blood culture rates vary from 30% to 78%, and about 25% to 59% of positive cultures identify the causative organism [18]. Some researchers recommend collecting two blood samples from different sites on the same day [21]. One study demonstrated a 68.6% positivity rate when three blood samples were collected at intervals of 30 minutes to 4 hours after biopsy, provided at least two samples showed growth of the same organism [22].

A diverse array of microorganisms can be isolated from cases of spondylodiscitis, with *Staphylococcus aureus* being the most frequent, accounting for nearly half of non-tuberculous cases [6]. The proportion of vertebral infections attributed to *Staphylococcus aureus *varies between 20% and 84% across bacterial vertebral infections [18]. *Escherichia coli, Proteus, Klebsiella,* and *Enterobacteriaceae *species are responsible for 7% to 33% of cases [18]. In the present study, infected disc material cultures demonstrated microorganism growth in five patients (17.8%, n=28), specifically *Staphylococcus aureus* in three patients, *Enterococcus faecalis* in one, and* Klebsiella* in another.

A bimodal age distribution pattern has been observed for spondylodiscitis, with peaks in those younger than 20 years and between 50 and 70 years [4]. The literature also tends to show a male predominance for this disease [9,23]. In the present study, one patient was 13 years old, and another was 22, while all other participants were in their forties or older, supporting the bimodal age pattern. Additionally, the study observed a 57.1% male predominance among subjects.

Infections of the spine can involve the vertebral bodies, intervertebral discs, paravertebral structures, and the spinal canal itself [24]. Numerous factors are known to predispose individuals to this condition, including skin and soft tissue infections, intravascular implants, genitourinary and gastrointestinal infections, respiratory or oral cavity infections, intravenous drug use, older age, HIV infection, and immunosuppressive comorbidities such as renal failure, hepatic cirrhosis, and rheumatological diseases [18]. More recently, the rising number of spinal infection cases has been attributed to advances in diagnostic techniques, an increase in healthcare-associated infections, and a growing vulnerable population [4].

Lumbar involvement is the most common in hematogenous pyogenic spondylodiscitis, followed by thoracic, cervical, and sacral regions [25]. This study found lumbar spine involvement in 62.5% of patients (n=15), dorsal spine involvement in 33.3% (n=8), and cervical involvement in 8.3% (n=2).

For cases of suspected pyogenic spondylodiscitis, obtaining preoperative blood and urine cultures before antibiotic administration is routine practice in some centres [18]. The responsible microorganism can be detected in both febrile and afebrile patients as well as those who are critically ill [26]. Blood culture remains a simple and cost-effective tool for identifying causative organisms in spondylodiscitis [27]. There is literature describing discordant findings between blood cultures and biopsy results [28]. Some studies suggest that collecting blood samples shortly after disc or vertebral biopsy improves diagnostic sensitivity [26]. Percutaneous or open biopsy yields positive results in 43% to 78% of cases. Four surgical samples are more likely to yield positive biopsy results [29,30]. The frequency of culture-negative spinal infection has been reported as ranging from 21% to 34% [31]. 

Antibiotic exposure before surgery is correlated with reduced diagnostic yield for microbiological testing; however, research also shows that higher tissue culture positivity does not necessarily correlate with a longer antibiotic-free interval in pyogenic vertebral osteomyelitis cases [32]. In two subjects, methicillin-resistant *Staphylococcus epidermidis* (MRSE) was detected in intraoperative blood cultures, but disc material cultures did not show bacterial growth, suggesting probable contamination from skin commensals.

There are certain limitations of the study, including the small sample size and the collection of only a single blood sample for culture in both pre- and perioperative periods, along with an arbitrary antibiotic-free interval of 48 hours and low positivity rates of blood culture. Although thorough tissue retrieval was performed, peripheral tissue sampling could not be guaranteed. Widespread use of antibiotics before admission may also have diminished the chances of pathogen identification.

Future investigations with larger sample sizes and multiple blood collections from different sites at several preoperative and intraoperative time points would provide more comprehensive data on the entry of microorganisms into the bloodstream during disc preparation in pyogenic spondylodiscitis. The results of the current study, however, fail to reject the null hypothesis. 

## Conclusions

The results of our study fail to reject the null hypothesis that manipulation of the infected disc material and preparation of the end-plate could be sources of bacteremia, as the intraoperative blood samples had no growth matching the infected disc material culture in the majority of cases. However, the small sample size, low positivity rate of culture, the collection of a single intraoperative blood sample, and a variable duration of antibiotic-free period could potentially affect the results. Thus, larger multi-centric studies involving patients without recent antibiotic exposure and including blood samples at multiple intervals in the immediate postoperative period are warranted to validate the true significance of intraoperative blood culture findings and to better define the risk of transient bacteremia during such procedures.
